# Interactions of the Nipah Virus P, V, and W Proteins across the STAT Family of Transcription Factors

**DOI:** 10.1128/mSphere.00449-20

**Published:** 2020-12-16

**Authors:** Timothy R. Keiffer, Michael J. Ciancanelli, Megan R. Edwards, Christopher F. Basler

**Affiliations:** a Center for Microbial Pathogenesis, Institute for Biomedical Sciences, Georgia State University, Atlanta, Georgia, USA; b Department of Microbiology, Icahn School of Medicine at Mount Sinai, New York, New York, USA; c Turnstone Biologics, New York, New York, USA; U.S. Centers for Disease Control and Prevention

**Keywords:** Nipah virus, STAT transcription factors, interferon

## Abstract

The Nipah virus (NiV) phosphoprotein (P) gene encodes four proteins. Three of these—P, V, and W—possess a common N-terminal domain but distinct C termini. These proteins interact with immune modulators. Previous studies demonstrated that P, V, and W bind STAT1 and STAT4 and that V also interacts with STAT2 but not with STAT3. The STAT1 and STAT2 interactions block interferon (IFN)-induced STAT tyrosine phosphorylation. To more fully characterize the interactions of P, V, and W with the STATs, we screened for interaction of each viral protein with STATs 1 to 6 by coimmunoprecipitation. We demonstrate that NiV P, V, and W interact with STAT4 through their common N-terminal domain and block STAT4 activity, based on a STAT4 response element reporter assay. Although none of the NiV proteins interact with STAT3 or STAT6, NiV V, but not P or W, interacts with STAT5 through its unique C terminus. Furthermore, the interaction of NiV V with STAT5 was not disrupted by overexpression of the N-terminal binding STAT1 or the C-terminal binding MDA5. NiV V also inhibits a STAT5 response element reporter assay. Residues 114 to 140 of the common N-terminal domain of the NiV P gene products were found to be sufficient to bind STAT1 and STAT4. Analysis of STAT1-STAT3 chimeras suggests that the P gene products target the STAT1 SH2 domain. When fused to GST, the 114-140 peptide is sufficient to decrease STAT1 phosphorylation in IFN-β-stimulated cells, suggesting that this peptide could potentially be fused to heterologous proteins to confer inhibition of STAT1- and STAT4-dependent responses.

**IMPORTANCE** How Nipah virus (NiV) antagonizes innate immune responses is incompletely understood. The P gene of NiV encodes the P, V, and W proteins. These proteins have a common N-terminal sequence that is sufficient to bind to STAT1 and STAT2 and block IFN-induced signal transduction. This study sought to more fully understand how P, V, and W engage with the STAT family of transcription factors to influence their functions. The results identify a novel interaction of V with STAT5 and demonstrate V inhibition of STAT5 function. We also demonstrate that the common N-terminal residues 114 to 140 of P, V, and W are critical for inhibition of STAT1 and STAT4 function, map the interaction to the SH2 region of STAT1, and show that a fusion construct with this peptide significantly inhibits cytokine-induced STAT1 phosphorylation. These data clarify how these important virulence factors modulate innate antiviral defenses.

## Abstract (precis)

How Nipah virus (NiV) antagonizes innate immune responses is incompletely understood. The P gene of NiV encodes the P, V, and W proteins.

## INTRODUCTION

Nipah virus (NiV) is a member of the *Henipavirus* genus in the *Paramyxoviridae* family. A zoonotic pathogen, NiV has been responsible for one major outbreak and a series of smaller outbreaks over the last 20 years (1–3). NiV infection presents with respiratory and neurological symptoms, in which encephalic symptoms can persist long term after initial infection clears (4). The NiV genome is approximately 18 kb in length and has six genes. The phosphoprotein (P) gene encodes four products, including the P protein from an mRNA that corresponds to the sequence of the full-length open reading frame. Due to a specific signal that directs non-template-encoded insertions into the mRNA by the viral polymerase, the V and W proteins are produced; these share a N-terminal domain with P, but have unique C termini (5–7). The P gene also encodes the C protein, which is produced from an internal open reading frame present in all P gene mRNAs (8). Although the P protein plays critical roles in viral RNA synthesis, V, W, and C have been demonstrated to modulate NiV pathogenesis. Wild-type NiV causes lethal respiratory disease after intranasal inoculation into ferrets (9). A V knockout of NiV was highly attenuated in ferrets, failing to cause disease or death. In contrast, a W knockout of NiV had an altered course of disease with animals succumbing to encephalitis rather than respiratory disease (9, 10). Disruption of C (or both C and W) resulted in attenuation and the altered disease course, leading to neurological disease (10).

Signal transducer and activator of transcription (STAT) proteins are transcription factors that play key roles in interferon (IFN) and cytokine signaling (11). There are seven STAT proteins in humans: STAT1, -2, -3, -4, -5a, -5b, and -6. These possess a common domain structure with various amino acids among the members. The domain structure includes an N-terminal domain (NTD), a coiled-coil domain (CCD), a DNA-binding domain (DBD), a linker domain (LD), an Src-2 homology domain (SH2), and a transactivation domain (12, 13). STATs are activated by JAK family tyrosine kinases. Tyrosine phosphorylated STATs form homo- or heterodimers and accumulate in the nucleus, where they activate the transcription of target genes (11, 14, 15).

The contribution of the C, V, and W proteins to NiV virulence reflects their capacity to carry out innate immune evasion functions (16–25). Among these activities are the capacities of P, V, and W to bind, through their common N-terminal domains, STAT1 and STAT2 (22, 26–30). These interactions can prevent IFN-α/β- and IFN-γ-induced phosphorylation of STAT1, prevent IFN-induced gene expression, and blunt the antiviral effects of IFNs (22, 26–30). The V protein, which is predominantly cytoplasmic at steady state, retains STAT1 and STAT2 in the cytoplasm through an interaction interface mapped to STAT1 residues 509 to 712 (22, 28). The W protein, which predominantly localizes to the nucleus, relocalizes STAT1 from the cytoplasm to the nucleus, inhibiting IFN induced gene expression and antiviral activity (30). NiV V residues 100 to 160 were previously demonstrated to be sufficient to bind and inhibit STAT1 (28). Deletions and mutations examined in the context of full-length P, V, and W identified amino acid residues 114 to 140 as critical for STAT1 interaction and inhibition (26, 27). Mutations of common N-terminal domain residues previously implicated in STAT1 binding also attenuate and modulate the course of NiV disease in ferrets, demonstrating that STAT1 binding influences pathogenesis (31). While STAT3 was previously demonstrated to not bind V, a recent proteomics study identified STAT4 as an interactor of both V and W, and point mutations in the N-terminal domain of either V or W prevented this interaction (32). This suggests that the common N-terminal domain of P, V, and W may mediate interaction with and inhibition of STAT4.

Here, we sought to more fully characterize the interaction of P, V, and W with each of the STAT family members, assessing binding specificity and function. These efforts demonstrate a common capacity of P, V, or W or the shared N-terminal domain to interact with and inhibit STAT1 and STAT4 function. We demonstrate that the common N-terminal domain residues 114 to 140 are sufficient to bind STAT1 and STAT4 and map the binding site for this peptide to the SH2 domain of STAT1. In addition, we identify interactions of NiV V with STAT5a and -5b and demonstrate that these involve the unique C-terminal region of V and that the interaction can impair STAT5b-mediated transcriptional activation without preventing STAT5b tyrosine phosphorylation. These findings cumulatively provide novel insights into the functions of key NiV virulence factors.

## RESULTS

### Interaction with STAT4 is conserved across NiV P, V, and W, whereas STAT5 specifically interacts with NiV V.

**FIG 1:**
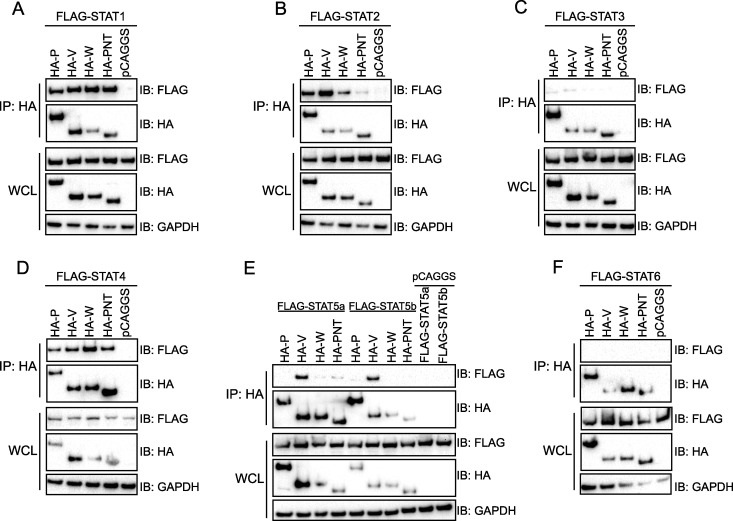
Interaction with STAT4 is conserved across NiV P, V, and W, whereas STAT5 specifically interacts with NiV V. A coimmunoprecipitation (co-IP) assay was performed on HEK293T cells transfected with plasmids encoding the indicated HA-tagged NiV P, V, W, and P N terminus (PNT) protein (2 μg) and FLAG-tagged STAT1 (A), STAT2 (B), STAT3 (C), STAT4 (D), STAT5a and -5b (E), and STAT6 (F) (2 μg). pCAGGS denotes the empty vector control. Co-IP was performed using anti-HA beads. Western blots for NiV and STAT protein expression in whole-cell lysates (WCL) and anti-HA bead elutions (IP: HA) were performed using anti-HA and anti-FLAG antibodies as indicated. Anti-GAPDH blots served as a loading control for the WCL. The immunoblots (IB) are representative of at least three independent experiments.

To provide a more complete assessment of NiV P gene product interactions with STAT family members, coimmunoprecipitation (co-IP) experiments were performed between hemagglutinin (HA)-tagged NiV P, V, and W and the common N terminus (P-NT) and FLAG-tagged STAT1, -2, -3, -4, -5a, -5b, and -6 (Fig. 1). All NiV proteins tested coprecipitated with STAT1, -2, and 4-, with STAT2 binding to P-NT appearing somewhat weaker that the STAT2 interactions with P, V, or W (Fig. 1A, B, and D). No interaction was detected with STAT3 or -6 (Fig. 1C and F). NiV V was unique in that it coprecipitated with STAT5a and -5b (Fig. 1E). Together, these co-IP assays indicate that the interaction with STAT1, -2, and -4 is conserved across the NiV P gene products, whereas NiV V makes an additional, specific interaction with STAT5.

### NiV V interacts with STAT5 via its C terminus and modulates STAT5 activity.

**FIG 2:**
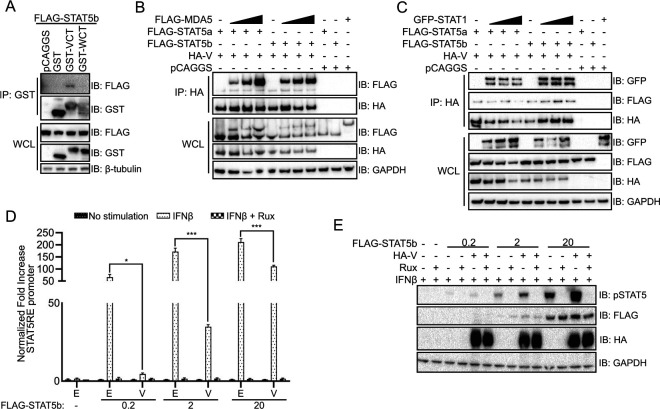
NiV V C-terminus interaction with STAT5 modulates its activity. (A) Co-IP was performed on 293T cells transfected with plasmids encoding FLAG-tagged STAT5b and empty vector control (pCAGGS) or GST alone or fused to the unique C terminus of NiV V or NiV W (GST-VCT and GST-WCT) using glutathione magnetic beads. Western blots were performed for FLAG and GST protein expression in whole-cell lysates (WCL) and bead elutions (IP: GST); the immunoblots (IB) are representative of two independent experiments. (B) A co-IP assay was performed using anti-HA beads on 293T cell lysate transfected with HA-tagged NiV V, FLAG-tagged STAT5a or -5b, and increasing concentrations of MDA5, as indicated. Western blots were performed for anti-HA and anti-FLAG. (C) A co-IP assay was performed as in panel B, on 293T lysates transfected with HA-tagged NiV V, FLAG-tagged STAT5a or -5b, and increasing concentrations of GFP-tagged STAT1. (D) 293T cells were transfected with increasing concentrations of FLAG-tagged STAT5b in 10-fold steps (0 to 20 ng), constitutively expressed *Renilla* luciferase reporter, STAT5 response element (STAT5RE)-firefly luciferase reporter plasmid, and HA-tagged NiV V, as indicated. Cells were treated with IFN-β and ruxolitinib, as indicated. The firefly luciferase signal was normalized to the *Renilla* luciferase signal, and the fold increase over mock-treated samples was determined. Error bars represent standard errors of four transfections performed in parallel. The experiment was performed three times. Statistical significance was determined by using a two-tailed *t* test (*; *P* < 0.05; ***; *P* < 0.001). E, empty vector control; V, transfection with HA-NiV V plasmid. (E) Western blot of the panel D luciferase assay samples treated with IFN-β and ruxolitinib, as indicated. Expression of HA-NiV V, Flag-STAT5b, and the phosphorylation status of STAT5b was assessed by Western blotting. IB, immunoblot.

The interaction of STAT5 with NiV V, but not P or W, suggests that the interaction is mediated by the unique C terminus of V. To assess whether this is the case, co-IP experiments were performed using glutathione *S*-transferase (GST) or GST-fused to the NiV V and W C termini (GST-VCT and GST-WCT, respectively). STAT5b coprecipitated with GST-VCT, but not with GST alone or GST-WCT (Fig. 2A). The unique C terminus of NiV V has previously been demonstrated to interact with the pattern recognition receptor MDA5, facilitating suppression of MDA5-mediated antiviral responses (19, 33). Given that the NiV V C terminus mediates interaction with STAT5, we used a competition co-IP to determine whether the exogenous expression of MDA5 disrupted STAT5 interaction with NiV V (Fig. 2B). Transfection of increasing concentrations of MDA5 (the upper band in Fig. 2B top panel) did not disrupt the interaction of NiV V with STAT5a or -5b (as shown in the lower band of Fig. 2B [top panel]). Similarly, overexpression of GFP-tagged STAT1, which binds the N terminus of NiV V, did not disrupt NiV V interaction with STAT5a or -5b (Fig. 2C). A slight decrease in STAT5a interaction at the highest concentration of STAT1 was detected, although this correlates with a decrease in the amount of NiV V precipitated, suggesting that STAT5 binding is separate from the STAT1 site (Fig. 2C). Together, this indicates that STAT5 interacts with NiV V through a C-terminal interface that differs from that used by MDA5.

To examine the impact of NiV V on the biological activity of STAT5, NiV V was tested in STAT5 response element (STAT5RE) reporter gene assays in which STAT5b was coexpressed, and IFN-β and the JAK1/JAK2 kinase inhibitor ruxolitinib were added as indicated (Fig. 2D). In the absence of an exogenous stimulus, no signal was detected. Since endogenous STAT5b is expressed at low levels in 293T cells, the STAT5b expression plasmid was transfected over a range of concentrations (34). Treatment with human IFN-β induced a signal corresponding to the amount of STAT5b transfected. The addition of NiV V significantly decreased the STAT5RE reporter induction, although the degree of inhibition decreased with higher STAT5b plasmid levels. Ruxolitinib completely abolished the STAT5RE signal, indicating that reporter expression was dependent on IFN-induced JAK activity (Fig. 2D). Together, these data indicate that the NiV V C terminus interacts with STAT5b, modulating its signaling activity.

Since NiV P, V, and W inhibit the phosphorylation of STAT1, we next sought to determine whether the NiV V interaction could affect STAT5b expression levels or phosphorylation status. Immunoblots were performed for total and phospho-STAT5b following IFN-β treatment in the presence or absence of ruxolitinib to test whether phosphorylation was JAK kinase dependent. IFN-β treatment induced STAT5b phosphorylation, with phosphorylation becoming more apparent as increasing amounts of STAT5b were expressed. Although the total STAT5b expression did not change in the presence of NiV V, phosphorylated STAT5b levels increased (Fig. 2E). This indicates that NiV V does not inhibit STAT5 phosphorylation and may in fact stabilize the phosphorylated form.

### Residues 114 to 140 make a major contribution to inhibition of STAT1 and STAT4.

**FIG 3:**
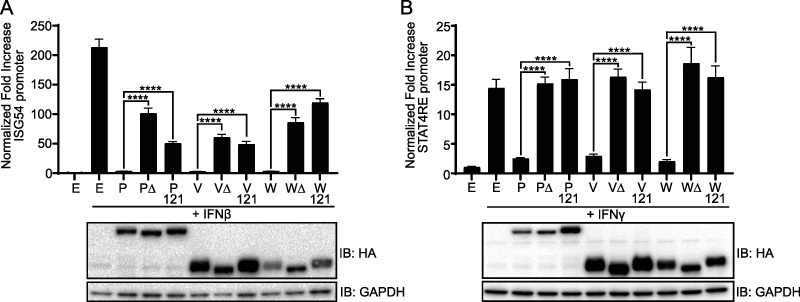
N-terminal residues 114 to 140 are necessary for efficient NiV P, V, and W inhibition of STAT1 and STAT4 activity. (A and B) HEK293T cells were transfected with an ISG54-promoter firefly luciferase reporter (A) or a STAT4-response element (STAT4RE) firefly luciferase reporter (B), constitutively expressed *Renilla* reporter and the indicated NiV P, V, or W wild-type (WT), the 114-140 deletion mutant (Δ), or a G121E point mutant (121) expression plasmid. E, empty vector control. Transfected cells were treated with IFN-β or IFN-γ, as indicated. At 24 h posttreatment, the firefly luciferase activity was normalized to the *Renilla* luciferase activity, and the fold increase over mock treatment was determined. Error bars represent standard errors of four transfections performed in parallel. The experiment was performed three times. Statistical significance was determined by using a two-tailed *t* test for the indicated samples (****; *P* < 0.0001). NiV protein expression was confirmed by Western blotting with anti-HA and anti-GAPDH. IB, immunoblot.

Deletion of the N-terminal residues 114 to 140 or mutation of select residues within this region of the P-gene derived proteins impairs their interaction with STAT1, decreasing inhibition of STAT1 activity (26). To determine whether these observations extend to STAT4, the abilities of wild-type, G121E (121), or 114-140 deletion mutant (Δ) forms of NiV P, V, or W to inhibit STAT activity were compared using ISG54 promoter and STAT4 response element (STAT4RE) firefly luciferase reporter assays. Cells were treated with IFN-β to induce ISG54 promoter activity, which occurs through STAT1-STAT2 heterodimers, or IFN-γ to activate the STAT4RE. In each of these assays, wild-type P, V, and W decreased the signal induced by IFN treatment to that of background, whereas both point and deletion mutants demonstrated a loss in the inhibition of reporter gene activity (Fig. 3).

### N-terminal residues 114 to 140 bind the STAT1 SH2 domain.

**FIG 4:**
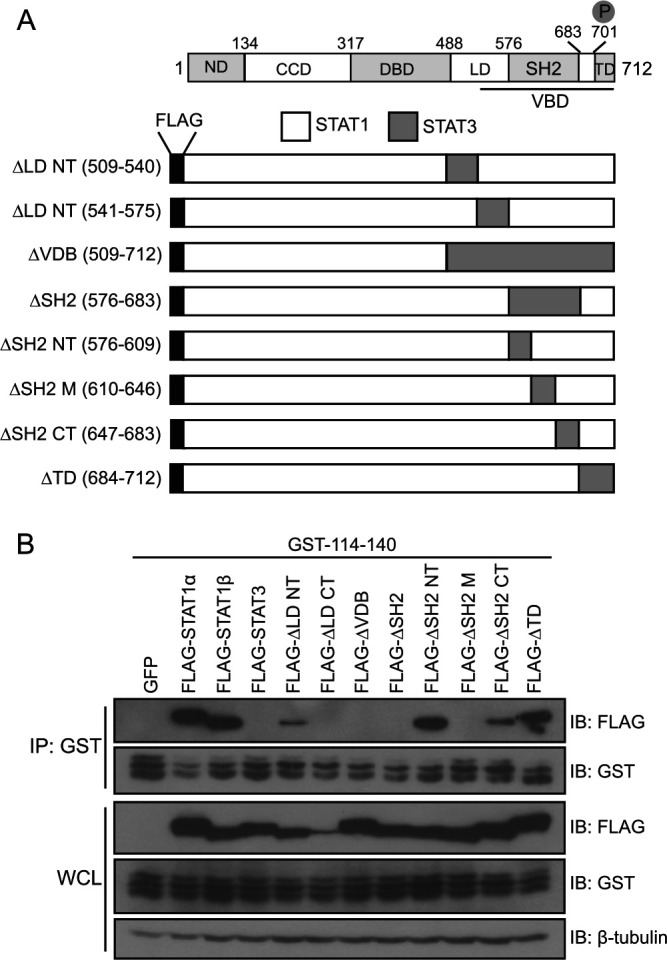
GST‐114-140 interacts with the SH2 domain of STAT1. (A) Schematic of STAT1β domains and the FLAG‐tagged STAT1/STAT3 chimeras. STAT1 comprises the N‐terminal domain (ND), coiled‐coil domain (CCD), DNA‐binding domain (DBD), linker domain (LD), Src‐homology 2 domain (SH2), and transactivation domain (TD). Larger domains are divided into N‐ and C‐terminal halves (NT and CT, respectively). (B) A co-IP was performed on HEK293T cells that were transfected with GFP, STAT1α, STAT1β, or STAT3 or the indicated STAT1/STAT3 chimeras and a plasmid encoding GST‐114-140. Co-IP was performed using glutathione magnetic beads. Western blotting was performed for FLAG and GST in whole-cell lysates (WCL) and bead elutions (IP: GST) as previously described. The immunoblots (IB) are representative of two independent experiments.

Binding studies using STAT1-STAT3 chimeras previously mapped the NiV V binding domain (VBD) to residues 509 to 712 of STAT1β, a splice variant of STAT1 which lacks 38 C-terminal residues found in STAT1α (28). This region spans several previously defined domains in STAT1: the linker domain (LD), the SH2 domain (SH2), and the transactivation domain (TD) (28). To better understand how residues 114 to 140 of NiV P interact with STATs, the binding site between the NiV P 114-140 peptide and STAT1 was mapped with STAT1-STAT3 chimeras (Fig. 4A). Because of the relatively large size of the linker and SH2 domains, multiple substitutions were made within these domains. Using a co-IP assay, GST-114-140 interacted with STAT1α and STAT1β but did not bind STAT3 (Fig. 4B), as was previously reported for NiV V (28). Substitution of the amino-terminal half of the linker domain of STAT1 (ΔLD NT) for the corresponding STAT3 region did not disrupt interaction with the 114-140 peptide, although it appeared to have a weaker interaction (Fig. 4B). The contribution of the carboxy-terminal linker domain of STAT1 to the interaction is difficult to assess due to poor expression of this construct (ΔLD CT). As expected, swapping the entire VBD in STAT1 for homologous regions in STAT3 disrupted interaction with residues 114 to 140, as did substitution of the entire SH2 domain. However, the residues at the amino and carboxy termini of the SH2 domain, 576 to 609 and 647 to 683, respectively, were not required for 114-140 interaction, whereas residues 610 to 646 (the middle of SH2, labeled SH2 M) were necessary. Lastly, replacement of the STAT1 TD with that of STAT3 did not impair interaction. Together, these data indicate that the binding by NiV 114-140 peptide requires amino acids 610 to 646 within the STAT1 SH2 domain.

### Fusion of N-terminal-derived peptides to GST is sufficient to mediate binding to and inhibition of both STAT1 and STAT4.

**FIG 5:**
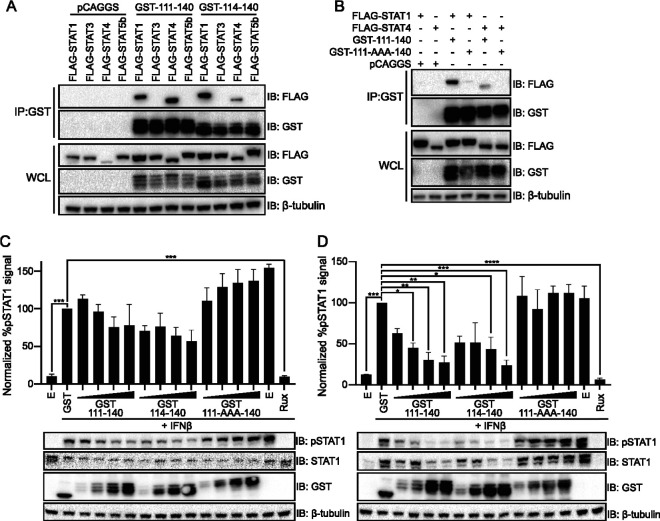
NiV P 111-140 peptide construct interacts with STAT1 and STAT4 and impairs STAT1 phosphorylation. (A) A co-IP was performed on HEK293T cells transfected with either an empty control plasmid (pCAGGS) or GST fusions to NiV P amino acid residues 111 to 140 or 114 to 140, and FLAG-tagged constructs of STAT1, STAT3, STAT4, or STAT5b, as indicated. Co-IP assays were performed with glutathione magnetic beads; Western blots were performed for FLAG and GST in whole-cell lysates (WCL) and elutions (IP: GST). The immunoblots (IB) are representative of two independent experiments. (B) HEK293T cells were transfected with either empty vector (pCAGGS) or GST-111-140 or GST-111-140 peptide with amino acid residues 114 to 116 replaced with alanines (111-AAA-140) or amino acid residues 114 to 140, derived from the N terminus of NiV P, and FLAG-tagged STAT1 or STAT4. Co-IPs were performed using glutathione magnetic beads; bead elutions (IP: GST) and lysates (WCL) were probed for FLAG and GST. The immunoblots (IB) are representative of two independent experiments. (C and D) HEK293T cells were transfected with increasing concentrations (2-fold dilutions, 32 to 250 ng) of plasmids expressing GST-111-140, GST-111-AAA-140, GST-114-140, or GST alone, as indicated. Cells were treated with IFN-β and ruxolitinib (rux) as indicated, for 30 min (C) or 24 h (D). Western blots were performed for GST, STAT1, and phosphorylated STAT1 (pSTAT1). The percent pSTAT1 was determined relative to the GST only control, which was set at 100%. Statistical significance, relative to the GST control, was determined by ANOVA with Dunnett’s multiple-comparison test (*, *P* < 0.05; **, *P* < 0.01; ***, *P* < 0.001; ****, *P* < 0.0001). The assay was performed in triplicate; error bars represent the standard errors for each triplicate. One representative Western blot is shown.

To further confirm the specificity of the interaction between the N terminus of the NiV P proteins and the STATs, co-IP experiments were performed with GST-111-140, GST-114-140, and STAT1, -3, -4, and -5b. As expected, both NiV P GST-111-140 and GST-114-140 coprecipitated STAT1 and STAT4, but not STAT3 or STAT5b (Fig. 5A). Prior experiments performed in the context of full-length P protein determined that mutation to alanine of residues 114 to 116 disrupts binding to STAT1 (26). We therefore made the equivalent mutations in the context of our GST-111-140 fusion (GST-111-AAA-140). This mutant did not interact with STAT4 and had a much weaker interaction with STAT1 (Fig. 5B).

The same GST-peptide constructs were assessed for inhibition of STAT1 phosphorylation. 293T cells transfected with plasmids that express GST or increasing concentrations of NiV P GST-111-140, GST-111-AAA-140, and GST-114-140 were stimulated with IFN-β for either 30 min or 24 h (h) to induce STAT1 phosphorylation (Fig. 5C and D). These two time points allowed us to test the impact of STAT1 shortly after its tyrosine phosphorylation was induced and, given that STAT1 expression is upregulated by IFN, to also assess the impact on longer term total and phospho-STAT1 levels (Fig. 5C and D). Expression of GST alone served as a control. We also included for comparison cells transfected with empty expression plasmid. The transfected cells were either mock treated or treated with IFN-β. The phosphorylated STAT1 signal is reported as a percentage relative to phosphorylated STAT1 in the GST alone control (Fig. 5C and D). After 30 min of stimulation with IFN-β, there was a trend toward decreased STAT1 phosphorylation as the concentration of GST-111-140 and GST-114-140 increased, although the change did not achieve statistical significance. After 24 h of stimulation, both the GST-111-140 and the GST-114-140 constructs inhibited IFN-induced upregulation of total STAT1 and led to significantly decreased levels of phospho-STAT1 (Fig. 5D). The mutant GST-111-AAA-140 peptide did not exhibit any capacity to block STAT1 phosphorylation after either short- or long-term stimulation (Fig. 5C and D). These data indicate that the 114-140 peptide is sufficient, at least when fused to a partner, to block IFN signaling.

## DISCUSSION

P, V, and W proteins play critical roles in the replication and virulence of NiV. The work presented in this study further clarifies and elaborates upon the mechanisms of NiV P, V, and W protein engagement of STAT-dependent signaling (22, 26, 28, 30, 35). STAT proteins are latent transcription factors that mediate the cellular response to a myriad of stimuli, including IFN (13). Upon binding to receptors, IFN activates Janus kinases (JAK1 and TYK2), which phosphorylate STAT molecules on conserved tyrosine residues. Type I IFNs (which include IFN-α/β) activate JAK1 and TYK2, resulting in the phosphorylation and heterodimerization of STAT1 and STAT2 (36–39). The STAT molecules translocate to the nucleus with IFN regulatory factor 9 (IRF9), where they induce transcription of IFN‐stimulated genes and upregulate an antiviral response (40–43).

Here, we determined the breadth of interaction between NiV P gene products and STATs (Fig. 1), demonstrating that in addition to the interaction with STAT1 and STAT2, the interaction with STAT4 is conserved across NiV P, V, and W. While STAT3 has been shown to lack interaction with NiV V, we demonstrate here that neither STAT3 nor STAT6 interact with any of the NiV P gene products (28). Notably, NiV V was found to form a specific interaction through its unique C terminus with STAT5 that was not shared by the other NiV P gene products (44). This interaction was not disrupted by exogenous expression of STAT1, an N-terminal interactor, or MDA5, a C-terminal interactor (Fig. 2B and C), suggesting that the interaction with STAT5 does not affect previously identified antagonist activity of NiV V (19, 33). Further study will be needed to define the exact NiV V-STAT5 interaction interface.

STAT5 is mostly studied in the context of development and cancer research (45, 46). However, STAT5 has been implicated in dendritic cell (DC) activation, and flaviviruses block STAT5 phosphorylation to counteract antiviral responses in DCs; these antiviral responses are activated by signaling through the type I IFN receptor, which promotes the maturation of DCs (47). In the case of NiV V, STAT5b activity was moderately decreased, and we did not detect a loss of STAT5b phosphorylation (Fig. 2D and E). There is precedent for this finding, since CD4^+^ cells from HIV-infected patients showed hyperphosphorylated STAT5, but phosphorylated STAT5 import into the nucleus after interleukin-7 stimulation was impaired (48). NiV infects certain immune cells such as monocytes and immature DCs where STAT5 is also expressed (49–55). Thus, it will be of interest to determine what effects NiV V has on STAT5 in these immune cell types and how this might affect cellular function(s).

Whereas the interaction between NiV V and STAT5 is mediated by the unique C terminus of NiV V (Fig. 2A), the interaction with STAT1 and STAT4, which share 61% identity, is via the common N-terminal residues 114 to 140 of the NiV P-gene proteins (56). Our interaction data with STAT1/STAT3 chimeras suggest that interaction with these STATs is mediated by the SH2 region (Fig. 4B). There is precedent for a viral protein to target this region of a STAT protein, since hepatitis C virus core protein also binds to the SH2 domain of STAT1, leading to decreased phospho-STAT1 levels (57). Future efforts should focus on further defining the binding interface between STAT1 and STAT4 with the N terminus of the NiV P-gene proteins since this could lead to development of strategies to block the immune-modulating functions of NiV P, V, and W.

The N-terminal residues 114 to 140 common to NiV P, V, and W were found to be important for the ability of these proteins to inhibit the activities of both STAT1 and STAT4 (Fig. 3A to C). Although numerous proteins from paramyxoviruses target IFN signaling (58), often through STAT1 (22, 29, 59–64), this is the first documented instance of proteins from a paramyxovirus inhibiting endogenous STAT4 activity. There is a clear benefit for NiV in suppression of IFN-induced cellular programs that block virus replication (65–67). Inhibition of STAT4 may also be relevant to IFN-α/β responses, because STAT4 can be activated by IFN-α in endothelial cells, a major target of NiV *in vivo* (68). Investigation into the contributions of NiV P, V, and W, along with their common N-terminal residues 114 to 140, can now be performed with NiV-specific reverse genetics systems (69). In a recent study of NiV disease progression in ferrets, a mutant virus where the STAT-binding activity of the NiV P gene products was disabled by mutation still exhibited a lethal phenotype, although the disease progression was shifted from a predominantly pulmonary disorder to a more neurological disorder (31). These data indicate that the STAT-binding functions of the common N-terminal domain modulate virulence. Whether the effects of the mutations are exclusively through STAT1 inhibition or whether inhibition of STAT4 contributes deserves further attention.

In addition to shedding light on NiV virulence factors, the data in this study also suggest applications for the N-terminal sequences that confer STAT binding and inhibition. Residues 114 to 140 (and residues 111 to 140) of the NiV P-gene proteins interact with both STAT1 and STAT4 (Fig. 5A and B) and block phosphorylation of endogenous STAT1 (Fig. 5C and D). These data introduce the possibility of using constructs derived from this peptide for anti-inflammatory therapeutics. STAT1 is critical to the antimicrobial immune response, but overactivation of STAT1 is a contributor to several inflammatory diseases such as asthma, celiac disease, and ulcerative colitis (70–72). STAT4 is a risk factor for several autoimmune diseases such as rheumatoid arthritis and systemic lupus erythematosus (73). Formulating STAT activity blockers based on Nipah virus proteins that selectively target these STATs may be advantageous since one of the recurring issues with current STAT treatments is lack of specificity (74). This peptide could potentially also guide rational design into additional therapeutics targeting overactive STAT1 and STAT4. Intracellular delivery would obviously be necessary for such a strategy to be effective.

## MATERIALS AND METHODS

### Cells and expression plasmids.

HEK293T (293T) cells were cultured at 37°C and 5% CO_2_ in Dulbecco modified Eagle medium supplemented with 10% fetal bovine serum, 1× antibiotic-antimycotic, and Plasmocin (Invivogen).

The pCAGGS-based plasmids that express HA-tagged NiV P, V, W, NiV P N-terminal residues 1 to 407 (PNT), FLAG-tagged MDA5, and GFP-tagged STAT1 were previously described (26, 75). Mutant NiV P G121E, V G121E, W G121E, P Δ(114-140), V Δ(114-140), and W Δ(114-140) were generated by PCR amplification. STAT1α (NM_007315), STAT1β (NM_139266), STAT3 (NM_139276), STAT4 (NM_003151), STAT5A (NM_001288718), and STAT5B (NM_012448) were amplified by RT-PCR from 293T cell RNA and cloned into pCAGGS with an N-terminal Flag tag. The STAT6 encoding plasmid pCMV-STAT6-IRES-Neo was a gift from Axel Nohturfft (Addgene, plasmid 35482 [http://n2t.net/addgene:35482]; RRID:Addgene_35482) and cloned into pCAGGS with an N-terminal Flag tag.

For GST pull-down studies, NiV P amino acid residues 111 to 140 and residues 114 to 140 and mutant 111-AAA-140, wherein residues 114 to 116 were replaced with alanine, were fused to an N-terminal GST in pCAGGS. STAT1/STAT3 chimeras were generated by overlapping PCR of the indicated regions from STAT1 and STAT3 and cloned into pCAGGS with a Flag tag.

### Coimmunoprecipitation experiments.

Equivalent amounts of NiV protein-expressing constructs, either HA or GST tagged, and STAT-expressing constructs were transfected into 10^6^ 293T cells using Lipofectamine 2000 (Thermo Fisher Scientific). Transfected cells were harvested at 24 h posttransfection and lysed with buffer containing 1% IGEPAL CA-630 (Sigma), 50 mM Tris (pH 8.0; Sigma), and 150 mM NaCl (Sigma) and supplemented with 1× cOmplete protease inhibitor cocktail (Roche). Lysates were cleared by centrifugation at 14,800 rpm for 10 min, followed by incubation with either anti-HA or glutathione magnetic beads (Pierce) at 4°C for at least 1 h. The magnetic beads were washed five times with lysis buffer, and bound proteins were eluted from beads using either a 3-fold excess of influenza HA peptide (Sigma) or by boiling under reducing conditions.

### ISG54-promoter and STAT4 response element assays.

293T cells (1 × 10^5^) were transfected with (i) the indicated reporter plasmid (50 ng), IFN-stimulated gene 54 (ISG54) promoter, or STAT4 response element (STAT4RE), all upstream of firefly luciferase; (ii) a constitutively expressing *Renilla* luciferase reporter plasmid (pRLTK; Promega) (10 ng); and (iii) wild-type or mutant NiV P, V, and W (50 ng) using Lipofectamine 2000. At 24 h posttransfection, the cells were treated with either 1,000 U/ml of human IFN-β (PeproTech) or 100 ng/ml of IFN-γ (PeproTech) for an additional 24 h. Firefly and *Renilla* luciferase levels were measured using a dual luciferase reporter assay system (Promega). IFN‐induced firefly luciferase signal was normalized to the constitutively expressed *Renilla* luciferase, and the fold activation over mock treatment was determined. The expression of wild-type and mutant P, V, and W proteins was confirmed by Western blotting.

### STAT5 response element assay.

293T cells (1 × 10^5^) were transfected with the STAT5 response element (STAT5RE; Promega) (50 ng), pRLTK (10 ng), STAT5b expression plasmid (0 to 20 ng), and a NiV V-expressing plasmid (50 ng). At 22 h posttransfection, STAT5b activity was induced by 1,000 U/ml IFN-β for an additional 24 h. Normalized luciferase activity was assessed and analyzed as described above.

### Endogenous STAT1 phosphorylation assay.

293T cells (1 × 10^5^) were transfected using Lipofectamine 2000 with increasing concentrations of plasmid expressing GST, GST-111-114, GST-114-140, or GST-111-AAA-140 (2-fold dilution; 32 to 250 ng); controls were transfected with 250 ng of pCAGGS. Cells were stimulated 22 h posttransfection with 1,000 U/ml IFN-β, as indicated, for either 30 min or 24 h. As an additional control, one sample was treated with IFN-β in the presence of a 5 μM concentration of the JAK1/JAK2 inhibitor ruxolitinib (SelleckChem) for the same time periods. After treatment, the cells were harvested and lysed with 1% IGEPAL CA-630–50 mM Tris–150 mM NaCl (pH 8.0) buffer containing 1× cOmplete protease inhibitor cocktail and 1× phosphatase inhibitor (Pierce). Samples were analyzed by Western blotting. The relative expression of phospho-(Y701) STAT1 was determined by normalizing its expression to the β-tubulin loading control using ImageJ software (76). Phosphorylation of STAT1 was plotted as a percentage of total phosphorylation of STAT1 in cells transfected with GST alone, which was set as 100%. Experiments were performed in triplicate (*n* = 3).

### Western blots.

Lysates were run on 4 to 12% Bis-Tris Plus polyacrylamide gels (Thermo Fisher) and transferred to polyvinylidene difluoride membranes (Bio-Rad). The membranes were probed with the indicated antibodies suspended in phosphate-buffered saline with 0.1% Tween 20 (PBS-T) in 5% milk (Bio-Rad) or, for phospho-blots, Tris-buffered saline with 0.1% Tween 20 (TBS-T) with 1× Blocker BSA solution (Thermo Scientific). Blots were developed by Western Lightning Plus ECL (Perkin-Elmer) and imaged on a ChemiDoc MP Imagining System (Bio-Rad). Either β-tubulin or GAPDH (glyceraldehyde-3-phosphate dehydrogenase) were used as loading controls.

Mouse anti-β-tubulin, anti-FLAG, anti-GAPDH, and anti-HA antibodies were purchased from Sigma-Aldrich. Mouse anti-STAT1 and anti-phospho(Y701F)-STAT1 were purchased from BD Transduction Laboratories. Rabbit anti-phospho-STAT5 and anti-GFP were purchased from Cell Signaling Technology. Mouse anti-GST was purchased from Abcam.

### Statistics.

Statistical significance was determined by using a two-tailed *t* test or analysis of variance (ANOVA) with Dunnett’s multiple-comparison test, as indicated in the figure legends (*, *P* < 0.05; **, *P* < 0.01; ***, *P* < 0.001; ****, *P* < 0.0001). Graphs were generated by using GraphPad Prism 8.
